# The Effectiveness of Web-Based Tailored Smoking Cessation Interventions on the Quitting Process (Project Quit): Secondary Analysis of a Randomized Controlled Trial

**DOI:** 10.2196/jmir.9555

**Published:** 2018-06-20

**Authors:** Bibhas Chakraborty, Raju Maiti, Victor J Strecher

**Affiliations:** ^1^ Centre for Quantitative Medicine Duke-NUS Medical School Singapore Singapore; ^2^ Department of Statistics and Applied Probability National University of Singapore Singapore Singapore; ^3^ Department of Biostatistics and Bioinformatics Duke University Durham, NC United States; ^4^ Department of Health Behavior and Health Education University of Michigan School of Public Health Ann Arbor, MI United States

**Keywords:** smoking cessation, number of quit attempts, tailored intervention, treatment regimen, Web-based intervention

## Abstract

**Background:**

Project Quit was a randomized Web-based smoking cessation trial designed and conducted by researchers from the University of Michigan, where its primary outcome was the 7-day point prevalence. One drawback of such an outcome is that it only focuses on smoking behavior over a very short duration, rather than the quitting process over the entire study period.

**Objective:**

The aim of this study was to consider the number of quit attempts during the 6-month study period as an alternative outcome, which would better reflect the quitting process. We aimed to find out whether tailored interventions (high vs low) are better in reducing the number of quit attempts for specific subgroups of smokers.

**Methods:**

To identify interactions between intervention components of smoking cessation and individual smoker characteristics, we employed Poisson regression to analyze the number of quit attempts. This approach allowed us to construct data-driven, personalized interventions.

**Results:**

A negative effect of the number of cigarettes smoked per day (*P*=.03) and a positive effect of education (*P*=.03) on the number of quit attempts were detected from the baseline covariates (n=792). Thus, for every 10 extra cigarettes smoked per day, there was a 5.84% decrease in the expected number of quit attempts. Highly educated participants had a 15.49% increase in their expected number of quit attempts compared with their low-educated counterparts. A negative interaction between intervention component *story* and smoker’s education was also detected (*P*=.03), suggesting that a high-tailored story given to highly educated people results in 13.50% decrease in the number of quit attempts compared with a low-tailored story.

**Conclusions:**

A highly individually tailored story is significantly more effective for smokers with a low level of education. This is consistent with prior findings from Project Quit based on the 7-day point prevalence.

## Introduction

Smoking is the leading preventable cause of death worldwide [1] and is associated with substantial economic burden [2]. Decades of research efforts have focused on evaluating effective computer-tailored smoking-cessation intervention programs [3,4]. These tailored programs are increasingly being delivered via technology-enabled platforms, for example, the internet [5,6], or more recently through mobile phone apps [7]. However, even with the support of modern technology, developing tailored smoking-cessation interventions is burdensome for both patients and health care providers. Hence, from a precision medicine perspective, it would be interesting to stratify a subgroup of smokers likely to benefit from tailored interventions. In this study, we used data from *Project Quit* [8,9], a randomized trial using Web-based tailored smoking cessation program and conducted secondary data analysis to identify this subgroup.

In modern quantitative precision medicine literature, the idea of personalizing treatments to individual patients is often operationalized as a *treatment regimen* [10-12]. Treatment regimen (TR) is a decision rule that takes available patient information as inputs to recommend some treatment. Constructing evidence-based (ie, data-driven) TR is typically a 2-step process consisting of hypothesis-generating data analysis and conducting a confirmatory trial [13]. An optimal TR estimated from existing data can be used to generate hypotheses on how an individual’s case history should guide treatment selection. These hypotheses can then be tested against a suitable control in a randomized controlled trial. Estimated optimal TR from Project Quit data analysis suggests that tailored interventions are most beneficial for smokers with low education and potentially detrimental to those with high education. Project Quit data analysis also suggests that tailored interventions do not have any impact on smokers with very high level of baseline addiction (those smoking >20 cigarettes/day).

Point prevalence is often a popular choice in assessing smoking cessation. In fact, Project Quit study was designed with a 7-day point prevalence as the primary outcome [8]. However, this outcome is based on subjects’ smoking status in a very limited time window (last 7 days) rather than the entire study period. An alternative outcome that better reflects the quitting process is the number of quit attempts over the entire study period [14-18]. It reflects participants’ involvement, or lack thereof, in the smoking cessation program. Although Project Quit study has collected data on number of quit attempts as a secondary outcome, this information has not yet been analyzed. Thus, we are focusing on this outcome in this study.

This study aims to identify a subgroup of smokers who are most likely to benefit from Web-based tailored behavioral interventions for smoking cessation. We will identify this subgroup based on their willingness and involvement in the quitting process, measured by the number of quit attempts during the study period. Such an approach will potentially allow health care researchers to use the limited public health resources more efficiently in shaping the health care policy.

## Methods

### Project Quit Trial

Project Quit was a Web-based smoking cessation program developed and conducted by the Center for Health Communications Research at the University of Michigan, Ann Arbor, and was funded by the National Cancer Institute (NCI), USA. The study protocol was reviewed and approved by the Institutional Review Board of each collaborating institution and of the University of Michigan in January 2004. The primary aim of the study was to identify and test the effects of 5 psychosocial and communication intervention components influencing smoking cessation [8]. The content of the Web-based intervention was based on cognitive-behavioral methods of smoking cessation, including an appeal to motives for quitting, stimulus control, self-efficacy enhancement, and suggestion for coping with tempting situations and emotions. Hypothetical success stories were employed within this overall paradigm.

Five intervention components (outcome expectations, efficacy expectations, success stories, message source, and message exposure) were studied in Project Quit. To screen multiple components, the study employed a *multiphase optimization strategy* [19] framework, implemented using a 16-cell (2^5-1^) fractional factorial design [20,21] in which each of the 5 intervention components were varied at 2 levels, high vs low. However, only 2 components, namely, success stories (hereafter referred to as *story*) and message source (hereafter referred to as *source*), were found to have significant effects on smoking cessation in previous analysis [8]. These 2 components were a priori hypothesized to have the strongest effect on smoking cessation. On the basis of these findings, we considered these 2 intervention components in our analysis. The intervention component *story* refers to success story received by study subjects from a hypothetical character who succeeded in quitting smoking. The *story* was varied at 2 levels—high vs low tailoring depth (ie, the degree to which the character in the story was tailored to subject’s baseline characteristics). Similarly, the component *source* refers to the source of Web-based behavioral counseling message received by subjects and was varied at 2 levels—high versus low level—of personalization. High-personalized source included photograph and supportive text from the health maintenance organization’s (HMO) smoking cessation team. It was written in a friendly language and included a signature from the team. In contrast, the low-personalized version included a photograph of a building, representing the HMO, and was impersonally written without a closing signature. Strecher et al [8] provided detailed description of these components, including examples of actual Web-based messages.

Adult participants were recruited from 2 HMOs—Group Health Cooperative (GHC), Seattle; and Henry Ford Health System (HFHS), Detroit; both these HMOs were affiliated with the NCI’s cancer research network. The study participants had a broad representation of ethnicity, gender, age, health status, and geography. Participants’ eligibility criteria included those who (1) had smoked at least 100 cigarettes in his or her lifetime, currently smoked at least 10 cigarettes per day, and had smoked in the past 7 days; (2) were seriously considering quitting in the next 30 days; (3) were 21 to 70 years; (4) were members of either GHC or HFHS; (5) had home or work internet access and an email account that they used at least twice weekly; (6) were not currently enrolled in other smoking cessation program(s) and not currently using pharmacotherapy for smoking cessation; and (7) had no medical contraindications for nicotine replacement therapy. A total of 1866 subjects participated in Project Quit. All subjects received free Web-based experimental smoking cessation program. Participants were randomized to receive either high or low personalized intervention, as described above. To pharmacologically assist them with smoking cessation, all participants, irrespective of their intervention group, also received a free 10-week supply of nicotine replacement therapy patches. Thus, this study allowed participants to focus on the cognitive-behavioral aspects of smoking cessation through combination of various intervention components.

The primary outcome of the study was the binary 7-day point prevalence in smoking cessation at 6 months following baseline assessment. During the 6-month evaluation survey, each subject was asked if she or he had smoked any cigarettes, even a puff, in the last 7 days. Subject who answered “yes” was marked as smoker and nonsmoker otherwise. In addition, data on the number of quit attempts in the past 6 months were collected as a secondary outcome, which is the focus of this study.

In addition to baseline covariates (age, gender, and race), Project Quit also collected variables deemed relevant for smoking cessation. These included (1) number of cigarettes smoked per day as a measure of baseline addiction; (2) the participant’s level of motivation to quit smoking as a predictor of smoking cessation [8]; (3) the participant’s level of education, which was hypothesized to interact with the intervention component *story* [8]; and (4) participant’s self-efficacy, a consistent predictor of subsequent health-related behavior change based on the social cognitive theory [22].

### Data Analysis

Of the 1866 subjects who enrolled in the Project Quit study, 1192 subjects responded to the question on the number of quit attempts. Of these responders, 792 subjects followed the study protocol by not using other smoking cessation aids or programs during the study. As the primary examination in Project Quit [8] utilized *per-protocol analysis* that only included subjects who did not violate study protocol, we used the same strategy to analyze the number of quit attempts in the 792 subjects.

To assess the potential presence of differential missingness across the intervention arms, we conducted chi-square test with 2 categorical variables—intervention arm (4 levels resulting from 2 intervention components, each varied at 2 levels) and nonresponse (2 levels, yes or no).

Baseline covariates considered in this analysis were age (continuous), gender (binary), race (3 levels, but handled by 2 dummy variables—race white and race black), cigarettes smoked per day (continuous), motivation (binary, high vs low, coded 1 or 0), self-efficacy (binary, high vs low, coded 1 or 0), and education (binary, ≤high school vs >high school, coded 0 or 1). The source and story levels were coded as 1 (high) and 0 (low), respectively.

We used the Poisson regression model to analyze the number of quit attempts. The Poisson regression model can be applied to settings where the outcome is a count-type variable with its expectation (mean) varying as a log-linear function of the covariates and intervention components. The model used in this analysis can be specified as log(E(Y|X_1_, X_2_, …, X_8_, A_1_, A_2_)) = β_0_ + β_1_ X_1_ + β_2_ X_2_ +…+ β_8_ X_8_+ (δ_0_ + δ_1_ X_7_)A_1_+ (η_0_ + η_1_ X_8_)A_2_, where *Y* denotes the number of quit attempts during the 6-month study period; X_i_, i=1, 2, …, 8, denote the baseline characteristics, viz, age, gender, race white, race black, cigarettes smoked per day, motivation, education, and self-efficacy, respectively; and A_1_ and A_2_ denote the intervention components story and source, respectively. The notation E(Y|X_1_, X_2_, …, X_8_, A_1_, A_2_) denotes the conditional expectation (conditional mean) of *Y*, given all the baseline covariates and intervention components. The unknown parameters (β_i_, i=0, 1, …, 8; δ_0_, δ_1_, η_0_, η_1_) in Poisson regression are estimated by the maximum likelihood method. We used open-source software R, version 3.2.3 [23] for the analysis.

Regression coefficients β_i_, i=1, …, 8, denote the main effects of the covariates X_i_, i=1, 2, …, 8; β_0_ denotes the model intercept; δ_0_ and η_0_ denote the main effects of intervention components A_1_ and A_2_, respectively; and finally, δ_1_ and η_1_ denote the preconceived interaction effect between X_7_ and A_1_ and that between X_8_ and A_2_, respectively. Instead of reporting the estimates of β_i_, we reported the corresponding adjusted incidence rate ratios, or simply the rate ratios (RRs). These quantities offer a more interpretable way to report results from a Poisson regression model (analogous to reporting odds ratios from a logistic regression model for binary data). Under the above setup, we defined RR for a covariate X_i_ as the ratio of the expectation of Y given that X_i_=1 and the expectation of Y given that X_i_=0 (for binary X_i_) or as the ratio of the expectation of Y given that X_i_=x+1 and the expectation of Y given that X_i_=x for some arbitrary value x (for continuous X_i_), given that other variables in the model (both covariates and interventions) are fixed. This RR can then be computed as the exponential transform of the regression coefficient (exp(β_i_)). RR measures change in the expected outcome when X_i_ increases by 1 unit (for continuous X_i_), or when X_i_ moves from 1 category to the other (for categorical X_i_) on a multiplicative scale.

Re-expression of intervention effects may further facilitate interpretation. The effect of a particular intervention component, say A_1_(story), can be expressed as E(Y|X_1_, X_2_, …, X_8_, A_1_=1, A_2_) – E(Y|X_1_, X_2_, …, X_8_, A_1_=0, A_2_) = (exp(δ_0_ + δ_1_ X_7_) – 1) E(Y|X_1_, X_2_, …, X_8_, A_1_=0, A_2_), which in turn can be interpreted as—given all other covariates are fixed, a highly tailored story (A_1_=1) increases the expected number of quit attempts by (exp(δ_0_ + δ_1_ X_7_) – 1)100% compared with the low-tailored story (A_1_=0). Similarly, for A_2_(source), it can be interpreted that a highly personalized source increases the expected number of quit attempts by (exp(η_0_+ η_1_ X_8_) – 1)100%, compared with the low-personalized source. Furthermore, for any of the baseline covariates, the effect of the *i*-th covariate can be expressed as (exp(β_i_) – 1)100%, i=1, …, 8.

We used the standard 5% alpha level to assess statistical significance in our analyses. Whenever appropriate, we also reported the 95% CIs of various effects. On the basis of the Poisson regression results, we then derived the corresponding TRs for recommending personalized smoking cessation interventions and drew decision trees to visually represent TRs.

We expected smokers’ baseline level of addiction, as measured by the number of cigarettes smoked per day (and found in our analysis results presented below), to influence the number of quit attempts. Therefore, once the Poisson regression analysis on the full data was completed, we divided the participants into 2 subgroups: (1) those who used to smoke less than or equal to the observed median of the number of cigarettes smoked per day and (2) those who used to smoke more than the observed median of the number of cigarettes smoked per day. We then repeated the Poisson regression analysis for each of the subgroups.

## Results

Before the primary data analyses, we examined potential differential missingness across the intervention arms and found no significant difference (*P*=.64).

### Descriptive Data Summary

The per-protocol participants’ baseline characteristics (n=792) are summarized in Table 1. These subjects had a mean age of 46.32 (SD 10.64) years. Of these, 60.6% (480/792) were female, 77.7% (615/792) were white, 12.3% (97/792) were African Americans, 63.4% (502/792) were highly educated, 53.2% (421/792) had high self-efficacy, and 45.7% (362/792) were highly motivated. On average, the participants used to smoke 21.51 (SD 8.94) cigarettes per day at baseline. With respect to randomized interventions, 51.4% (407/792) subjects received a highly tailored story, and 49.9% (395/792) subjects received a highly personalized source. During the 6-month study period, 93.3% (739/792) participants attempted to quit at least once. The number of quit attempts varied from 0 to 10, and the mean quit attempt was 2.74 (SD 2.50) times.

**Table 1:** Participant characteristics. Descriptive summary refers to mean (SD) for continuous characteristics and frequency (percentage) for categorical variables.

Participant characteristics		Descriptive summary (n=792)
Age in years, mean (SD)		46.32 (10.64)
**Gender, n (%)**		
	Female	480 (60.6)
**Race, n (%)**		
	African American	97 (12.3)
	White	615 (77.7)
	Other	80 (10.1)
**Education, n (%)**		
	>High school	502 (63.4)
	≤High school	290 (36.6)
Number of cigarettes smoked per day, mean (SD)		21.51 (8.94)
**Motivation, n (%)**		
	High	362 (45.7)
	Low	430 (54.3)
**Self-efficacy, n (%)**		
	High	421 (53.2)
	Low	371 (46.8)
**Story, n (%)**		
	Deeply tailored	407 (51.4)
	Low-tailored	385 (48.6)
**Source, n (%)**		
	Highly personalized	395 (49.9)
	Low-personalized	397 (50.1)

### Poisson Regression Results

The estimated Poisson regression coefficients, z-scores, RR values along with their 95% CIs, and corresponding *P* values are reported in Table 2. After adjusting for relevant covariates and treatment components, only the number of cigarettes smoked per day at baseline (RR=0.994; 95% CI 0.989-0.999; *P*=.03), education (RR=1.155; 95% CI 1.018-1.311; *P*=.03), and education-by-story interaction (RR=0.825; 95% CI 0.692-0.985; *P*=.03) were significant. This means that when all other covariates are fixed in the model, for every extra cigarette smoked per day at baseline, the expected number of quit attempts in the 6-month study period changes by a multiplicative factor of 0.994. In other words, for every 10 extra cigarettes smoked per day at baseline, there is a 5.84% decrease in the expected number of quit attempts over 6 months. On the other hand, there is a 15.49% increment in the expected number of quit attempts for highly educated participants (*P*=.03), as compared with those with low education.

**Figure 1:**
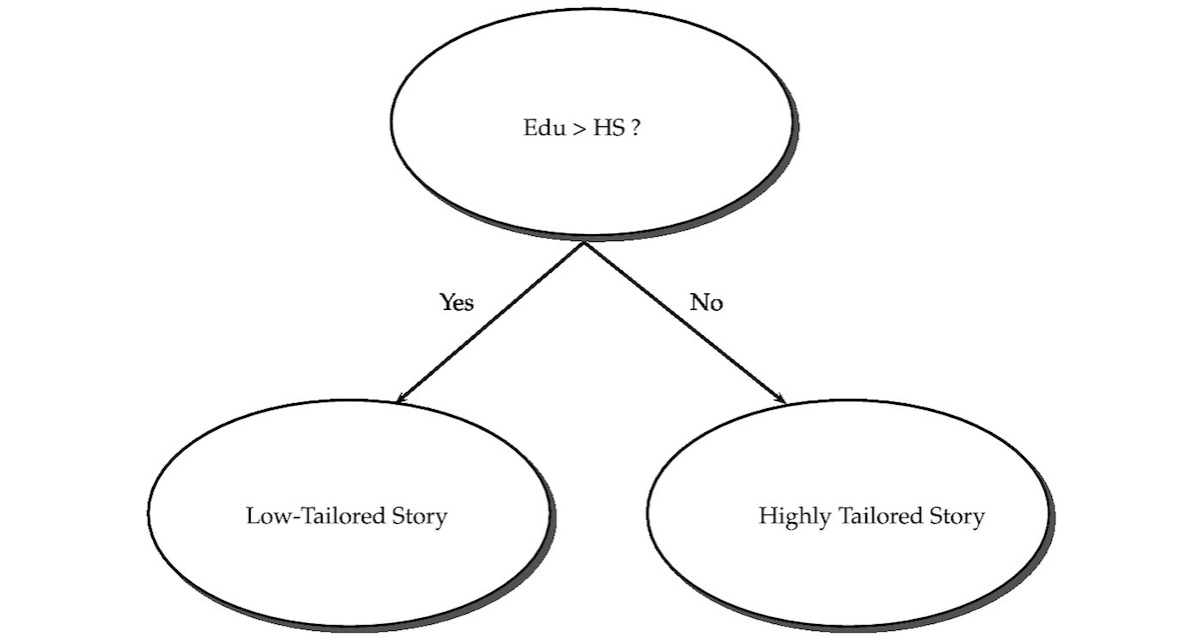
An estimated treatment regimen to recommend personalized smoking cessation intervention for the whole population. Edu: education; HS: high school.

Interaction between education and story is interpreted differently from the main effects of individual covariates. For this scenario, the main effect and interaction effect should be interpreted jointly. Table 3 shows the effects of both high- and low-tailored stories on the 2 levels of education. Giving a high-tailored story to a highly educated smoker results in a 13.5% decrease in the number of quit attempts compared with a low-tailored story *(P*=.03). However, the result is completely reverse for the low-educated group, where a high-tailored story given to a low-educated person increases the expected number of quit attempts by 4.8% compared with a low-tailored story. On the basis of this result, we derived a TR that recommends personalized smoking cessation interventions (high-tailored story for low-educated subjects and low-tailored story for those who are highly educated). A decision tree to visualize this TR is shown in Figure 1.

We have shown that smokers’ baseline level of addiction, as measured by the number of cigarettes smoked per day, has a negative impact on the number of quit attempts. Using the observed median of 20 cigarettes smoked/day as a threshold, we further divided the participants into 2 subgroups: (1) those who used to smoke ≤20 cigarettes/day and (2) those who used to smoke >20 cigarettes/day. We found that severe smokers (>20 cigarettes/day at baseline) were not influenced by any intervention components. However, less severe smokers with lower education were more influenced by the highly tailored story, which is similar to the whole group of smokers in the study. Results from the Poisson regression analyses for the less severe subgroup of smokers (≤20 cigarettes/day at baseline) are shown in Tables 4 and 5, and can be interpreted in a similar fashion as above.

**Figure 2:**
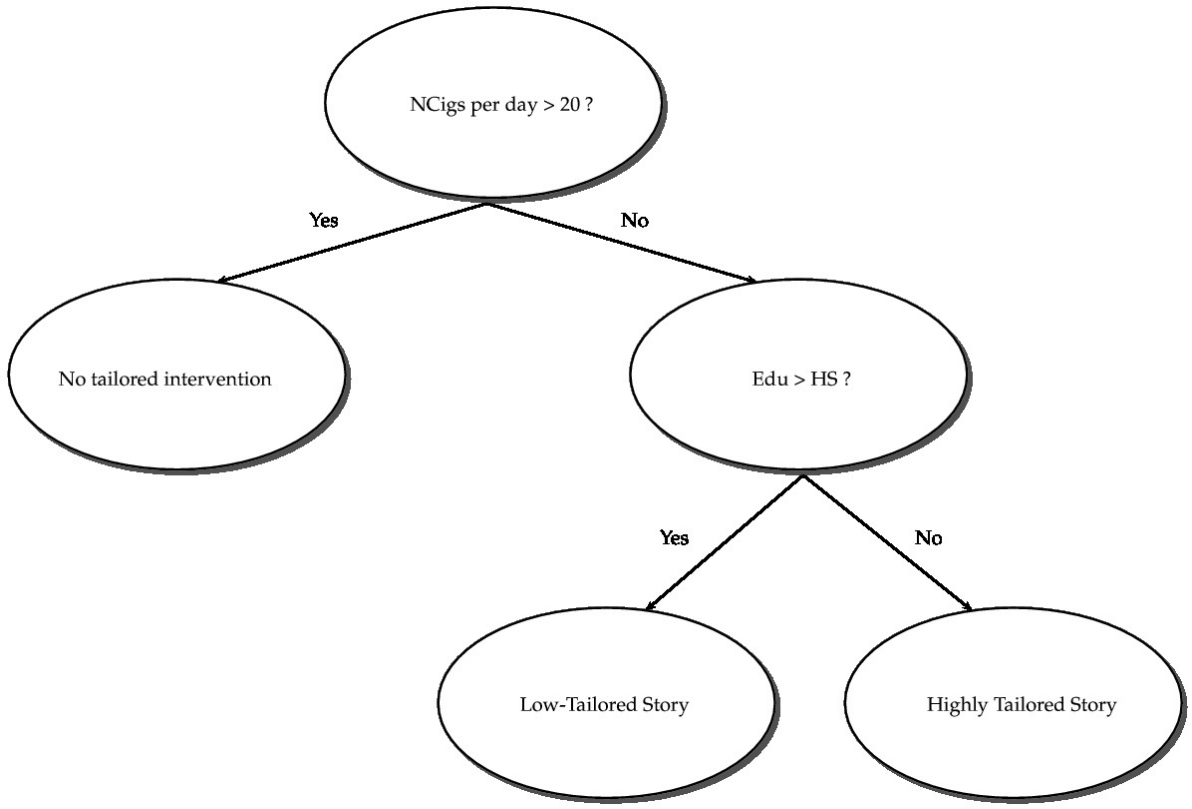
An estimated treatment regimen to recommend personalized smoking cessation intervention for smokers who smoked less than or equal to 20 cigarettes per day at baseline. Edu: education; HS: high school; NCigs: number of cigarettes smoked.

In addition to the effects that were significant in the full dataset, the main effects of self-efficacy and the intervention component story also came out significant in this subgroup analysis. Figure 2 shows the decision tree for the associated TR.

**Table 2:** Summary results of the Poisson regression model for the number of quit attempts outcome (n=792).

Variable	Regression parameter estimate	Z-score	Adjusted rate ratio (95% CI)	*P* value
Age (years)	0.001	0.662	1.001 (0.997-1.005)	.51
Gender (male)	0.070	1.572	1.073 (0.983-1.171)	.12
Race (dummy for white)	−0.041	−0.577	0.959 (0.833-1.105)	.56
Race (dummy for Black)	0.134	1.504	1.143 (0.960-1.361)	.13
Number of cigarettes smoked per day (NCigs per day)	−0.006	−2.185	0.994 (0.989-0.999)	.03^a^
Motivation	0.055	1.148	1.056 (0.962-1.160)	.25
Education	0.144	2.230	1.155 (1.018-1.311)	.03^a^
Self-efficacy	−0.054	−0.840	0.948 (0.836-1.074)	.40
Story	0.047	0.643	1.048 (0.908-1.209)	.52
Source	−0.082	−1.285	0.921 (0.813-1.044)	.20
Story × education	−0.192	−2.125	0.825 (0.692-0.985)	.03^a^
Source × self-efficacy	0.080	0.926	1.084 (0.914-1.284)	.36

^a^Denotes *P*<.05.

**Table 3:** Estimated intervention effect of story, expressed as a percentage change in the expected number of quit attempts, stratified by education level, mathematically expressed as (exp(δ_0_ + δ_1_ Edu) – 1)100% (n=792).

Education	Estimate (95% CI)
>High school (Edu=1)	−13.50 (−22.58 to −4.42)
≤High school (Edu=0)	4.798 (−10.17 to 19.76)

**Table 4:** Summary results of the Poisson regression model for participants who smoked less than or equal to 20 cigarettes per day (n=546).

Variable	Estimate	Z value	Adjusted rate ratio (95% CI)	*P* value
Age (years)	0.002	0.676	1.002 (0.997-1.006)	.50
Gender (male)	0.083	1.539	1.087 (0.972-1.202)	.12
Race white	−0.148	−1.813	0.862 (0.724-1)	.07
Race black	−0.051	−0.504	0.950 (0.762-1.139)	.61
Number of cigarettes smoked per day (NCigs per day)	−0.014	−2.108	0.986 (0.973-0.999)	.03^a^
Motivation	0.095	1.687	1.099 (0.978-1.221)	.09
Education	0.256	3.142	1.291 (1.085-1.497)	.002^a^
Self-efficacy	−0.159	−2.131	0.853 (0.729-0.978)	.03^a^
Story	0.189	2.022	1.208 (0.987-1.428)	.04^a^
Source	−0.077	−1.028	0.926 (0.790-1.062)	.30
Story × education	−0.414	−3.700	0.661 (0.516-0.806)	<.001^a^
Source × self-efficacy	0.052	0.499	1.053 (0.839-1.267)	.62

^a^Denotes *P*<.05.

**Table 5:** Estimated intervention effect of story, expressed as a percentage change in the expected number of quit attempts, stratified by the education level for the persons who smoked less than or equal to 20 cigarettes per day (n=546).

Education	Estimate (95% CI)
>High school (Edu=1)	−20.20 (−29.93 to −10.47)
≤High school (Edu=0)	20.76 (−1.32 to 42.83)

## Discussion

### Principal Findings

This study aimed to stratify smokers who are likely to benefit from tailored smoking cessation intervention programs and those who are not. This will allow us to develop personalized smoking cessation interventions. Outcomes of this study can potentially help policy makers to allocate limited public health resources to target subgroups of smokers who are more likely to be successful from tailored smoking cessation interventions. This study analyzed existing data from a large randomized, Web-based smoking cessation trial (Project Quit) to answer the above research question.

In Project Quit, Strecher et al [8] previously studied the impact of 5 Web-based intervention components on the 7-day point prevalence. Using a multivariable logistic regression model, they found that 2 intervention components, namely, story and source, have significant effect on smoking cessation at 6 months. Furthermore, they showed that participants with lower education were more influenced by highly tailored stories, and a highly personalized source had marginally greater impact on smoking cessation in participants with higher self-efficacy. However, 1 drawback with the 7-day point prevalence is that it does not take into account the quitting process over the entire study period. Instead, it only focuses on smoking behavior in a limited time window at the end of the 6-month follow up. Our investigation was designed to specifically overcome this limitation by considering the number of quit attempts during the whole study period as the outcome of interest and examine whether similar effects still hold. The number of quit attempts quantified participants’ willingness and involvement in the smoking cessation process throughout the study period. Our study incorporated 2 findings from prior analysis of Project Quit [8] into this analysis. First, of the 5 intervention components from the original trial, we considered only 2 (story and source) into our model because of their significant effects (remaining components were insignificant). Second, based on the a priori hypothesis and data analysis from Strecher et al [8], we only included 2 interaction effects—one between story and education, and another between source and self-efficacy.

We found that participants with lower education (high school graduates or less) were positively influenced by a high-tailored story to quit smoking, whereas those with higher education were better off with a low-tailored story. Our findings are consistent with those from Strecher et al [8], who found similar effect modification, albeit in the context of the 7-day point prevalence. Findings on the low education group are not surprising as participants in this category can easily associate themselves with fictitious characters in the story that are tailored to them (socioeconomically or otherwise), as opposed to untailored characters. Such association allows the low-educated subjects to “transport” themselves into the story, thus resulting in successful smoking cessation. Strecher et al [8] suggested that the extent of being “transported” has a strong influence on persuasion, which in particular applies to smokers’ attempt and behavior to quit [24,25]. In contrast to Strecher et al [8], we did not find any significant interaction between source and self-efficacy in influencing the number of quit attempts. We speculate that such discrepancy may be due to different outcome under consideration or the smaller sample size in this investigation.

Here, we summarize the strengths of this study that was designed to address gaps in the extant literature on smoking cessation. First, ours is the first analysis of quit attempts data from Project Quit. This will potentially shed new light on smokers’ quitting process experience while participating in a Web-based smoking cessation program. Second, although analysis of quit attempts data are available in the literature [14-18], they are based on observational cohort studies. Our study is the first to analyze quit attempts data from a randomized trial. Third, we utilize TR as a perspective from the precision medicine literature to help better understand the type of smokers who will benefit from tailored intervention in optimizing their quitting effort. The results suggest that smokers with low education are more likely to benefit from tailored interventions. This is consistent with prior findings based on the 7-day point prevalence data [8,20,26]. Thus, this analysis validates the significance of number of quit attempts as an alternative to the commonly used point prevalence outcome. Finally, from a methodological perspective, number of quit attempts is a count-type variable rather than a continuous measurement or binary indicator. Hence, we employed Poisson regression to analyze the dataset.

There are a few limitations in our study. First, because the number of quit attempts was a secondary outcome in Project Quit, this variable had higher rate of missingness compared with the primary outcome of the 7-day point prevalence. For simplicity and easy interpretation, we only conducted a complete-case analysis. One could potentially employ missing data analysis techniques (eg, multiple imputation) to impute the missing values before conducting the analysis. However, as we did not find any evidence of differential missingness across the intervention arms, we argue that the missingness in the current data is mostly noninformative. Thus, data imputation techniques would not offer much benefit over a complete-case analysis. Second, the intervention components in the original study were designed to influence the 7-day point prevalence. One could conceive other potential intervention components not studied in Project Quit, which may potentially better influence smokers’ involvement in their difficult journey toward quitting and their number of quit attempts in particular. We believe that new studies specifically designed to understand the impact of tailored interventions on quit attempts are necessary to answer such questions. Third, the number of quit attempts is a self-reported outcome over a reasonably long period. As it is unrealistic for the participants to remember their exact number of quit attempts in the past 6 months, this variable may have recall bias. However, this concern can be addressed in the current era of mobile health and sensor technologies. New-generation studies should employ mobile apps and wearable devices to capture quit attempts data more accurately and thus minimize measurement errors.

### Conclusions

In this study, we aimed to shed new lights on the impacts of Web-based tailored psychosocial and communication intervention components on smoking cessation. Using data from a randomized Web-based trial, we examined the number of quit attempts during a 6-month study period. We also investigated how these impacts are modified by individual characteristics. Collectively, we aimed to identify subgroups of smokers who would successfully benefit from Web-based tailored interventions. We found that highly individually tailored story is significantly more effective for smokers with low education (high school graduate or less) compared with those with higher education (at least some college exposure). Our findings can provide evidence and potentially help policy makers to utilize limited public health resources to cease smoking in low-educated smokers. Nevertheless, we must cautiously note that the number of quit attempts in this study is self-reported, and thus subjected to recall bias. Future studies that incorporate sensor and/or mobile technologies to collect precise data on quit attempts are clearly warranted.

## Abbreviations

GHC: Group Health Cooperative
HFHS: Henry Ford Health System
HMO: health maintenance organization
NCI: National Cancer Institute
NCig: number of cigarettes smoked per day
RR: rate ratio
TR: treatment regimen
